# Anomalous Retro-Aortic Left Circumflex Artery Identified During Transcatheter Patent Foramen Ovale Closure: Procedural Implications

**DOI:** 10.1016/j.jaccas.2026.109546

**Published:** 2026-09-19

**Authors:** Khalid Alharbi, Wail Alkashkari, Abdullah Alsaiedi, Jamilah Alrahimi, Raneem Fallatah, Anas Merdad, Rasha Al-Bawardy

**Affiliations:** a King Abdullah International Medical Research Center, Jeddah, Saudi Arabia; b Department of Cardiac Sciences, King Faisal Cardiac Center, National Guard Hospital, Jeddah, Saudi Arabia; c College of Medicine, King Saud Bin Abdulaziz University for Health Sciences, Jeddah, Saudi Arabia

**Keywords:** coronary artery, coronary artery anomaly, myocardial ischemia, patent foramen ovale, transcatheter closure

## Abstract

**Background:**

Coronary artery anomalies are uncommon; certain origin and courses may be more vulnerable to compression by septal occluder devices and warrant careful evaluation.

**Case Summary:**

A 43-year-old man with recurrent transient ischemic attacks and embolic stroke was referred for transcatheter patent foramen ovale (PFO) closure. Coronary angiography demonstrated absence of the left main coronary artery with an anomalous origin of the left circumflex artery from the right coronary artery. Transesophageal echocardiography confirmed a retro-aortic anomalous coronary sign. After device deployment but before the release, repeat right coronary artery angiography confirmed preserved TIMI III flow in the anomalous left circumflex artery without evidence of compression.

**Discussion:**

Recognition of coronary anomalies before PFO closure is essential. When a retro-aortic coronary course is present, coronary flow should be reassessed before final device release to exclude mechanical compression.

**Take-Home Message:**

Comprehensive coronary assessment is critical during PFO closure when anomalous coronary anatomy is present.

## Graphical Abstract

**Figure:**
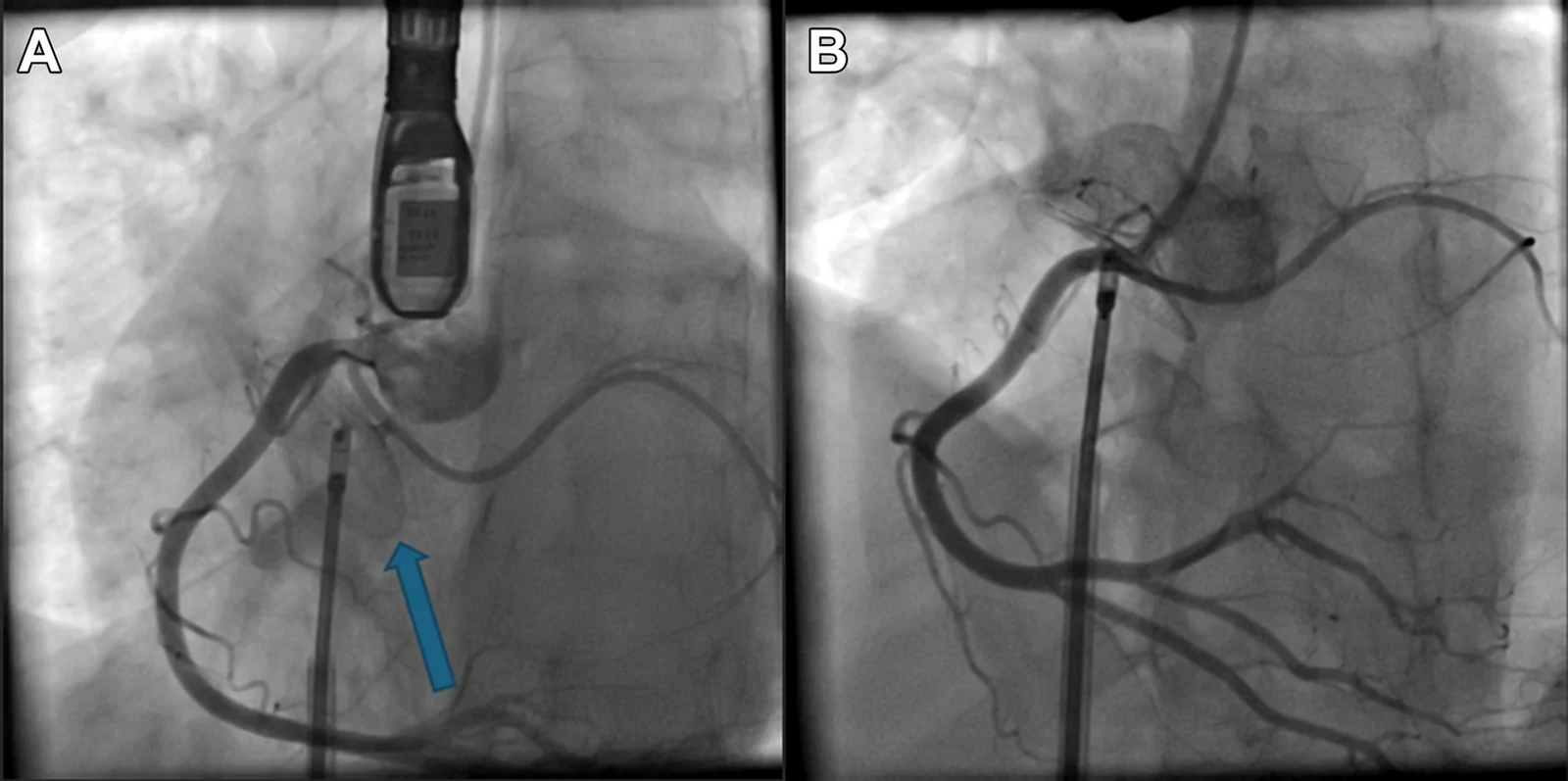

Congenital coronary artery anomalies with retro-aortic course were historically thought to be benign, with prevalence of approximately 1%.^1^ There are many types of coronary anomalies, with the most common type described as an anomalous origin of the left circumflex artery (LCx) from the right sinus of Valsalva.^1^

**Take-Home Message**

• Comprehensive coronary assessment is critical during PFO closure when anomalous coronary anatomy is present.

The coexistence of an anomalous LCx and patent foramen ovale (PFO) presents a unique clinical challenge. Although the true prevalence of this association is unknown, it has been reported in 2 of 75 patients undergoing transcatheter PFO closure.^2^ Detection of anomalous coronary arteries at the time of PFO closure raises 2 major concerns: the potential risk of sudden cardiac death and the possibility of mechanical compression by the closure device. Although many routes of anomalous coronaries are not typically of concern, some do carry that risk such as retro-aortic LCx.^3^

We report a rare case of an incidental anomalous LCx originating from the right coronary artery (RCA) identified during a transcatheter PFO closure, and the procedural steps undertaken to prevent mechanical compression.

## History of Presentation

A 43-year-old man with history of recurrent transient ischemic attacks and a previous embolic left middle cerebral artery stroke was referred for PFO closure.

## Past Medical History

The patient's medical history includes protein S deficiency and Factor V Leiden mutation.

## Differential Diagnosis

In young adults presenting with stroke or transient ischemic attack, the differential diagnosis is broader than in older patients and includes both vascular and nonvascular causes. Important vascular etiologies include cerebral artery dissection, cardioembolic in the presence of PFO, inherited and acquired thrombophilias, vasculitis, and some rare genetic disorders. Traditional vascular risk factors, including hypertension, diabetes, smoking, and dyslipidemia, also should be assessed for early atherosclerosis.

## Investigations

Brain imaging excluded carotid and intracranial vascular disease. Extended Holter monitoring showed no evidence of atrial fibrillation. Transthoracic echocardiography with bubble study confirmed an intra-atrial shunt consistent with a PFO.

## Management and Outcomes

Following multidisciplinary heart team discussion, transcatheter PFO closure was recommended. The patient was brought to the cardiac catheterization laboratory. A diagnostic coronary angiogram was first performed to evaluate coronary artery disease given the patient was >40 years of age. This revealed absence of a left main coronary artery and an anomalous LCx originating from the RCA with a retro-aortic course. No obstructive coronary artery disease was identified.

PFO closure was performed via right femoral venous access under transesophageal echocardiographic guidance. Imaging demonstrated a retro-aortic anomalous coronary sign (Figures 1 and 2, Video 1), consistent with angiographic findings (Figure 3).

**Figure 1:**
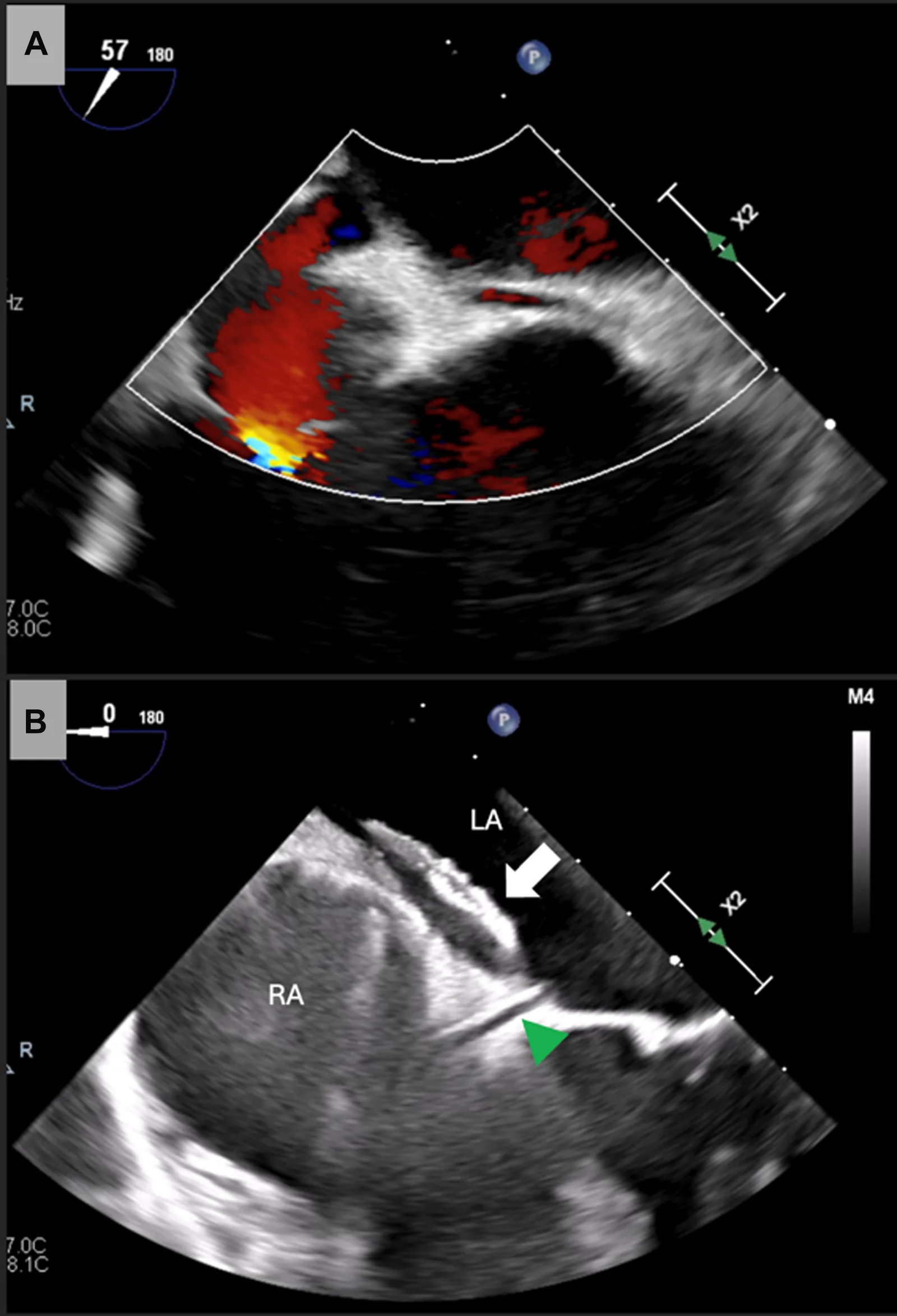
Mid-Esophageal Short-Axis Views During Patent Foramen Ovale Closure (A) Color Doppler shows patent flow, confirming this is a vascular structure—consistent with the retro-aortic anomalous coronary sign (a tubular echogenic structure behind the aortic root, suggesting a retro-aortic course of an anomalous coronary artery, which is usually considered a benign course). (B) Left atrial disc of the closure device (white arrow) is in proper position. A highly echogenic tubular structure is seen posterior to the aorta in the atrial portion of the atrioventricular groove (green arrowhead). LA = left atrium; RA = right atrium.

**Figure 2:**
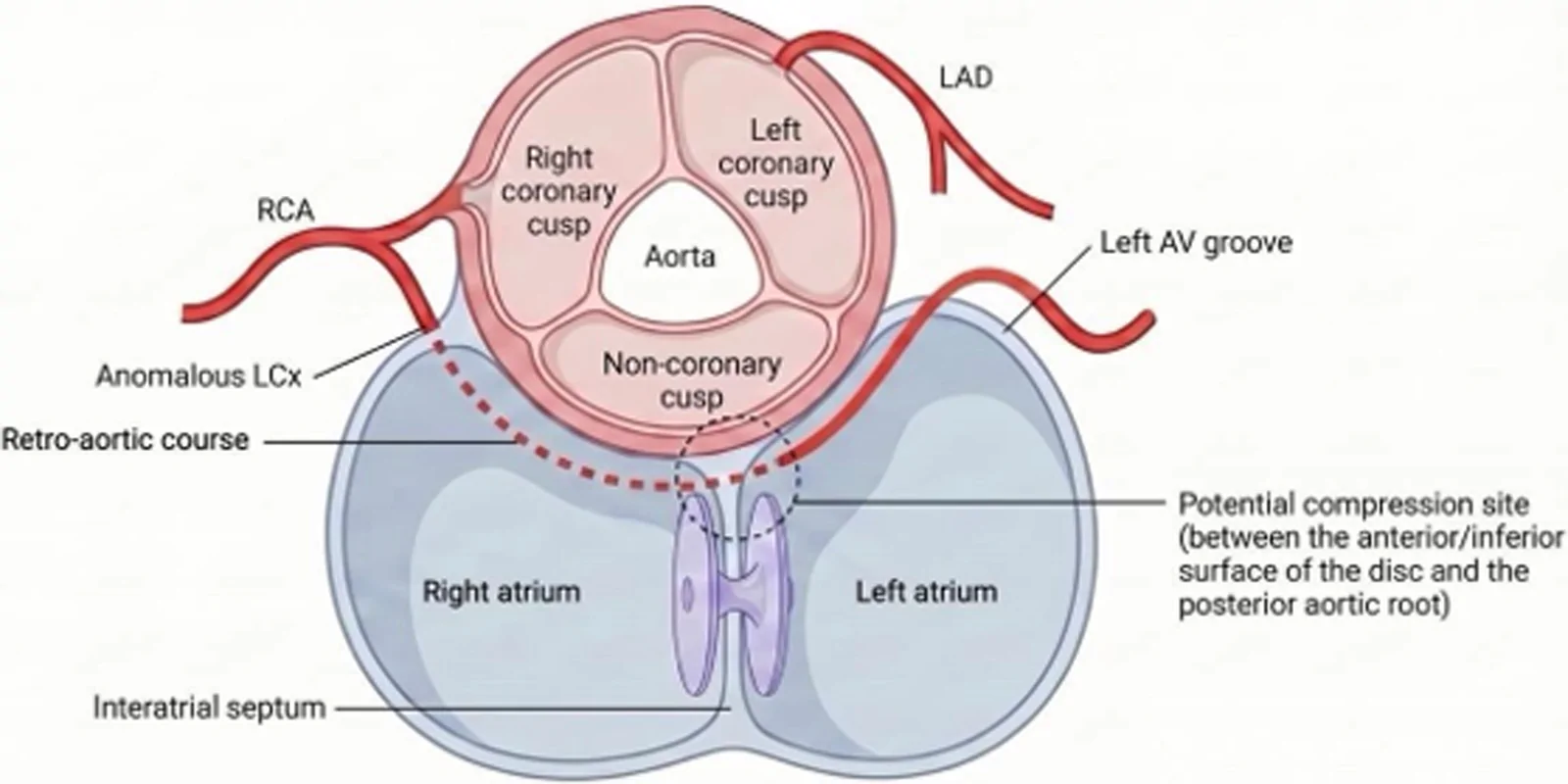
A Visual Illustration Showing the Retro-Aortic Course of An Anomalous Left Circumflex That Can be Potentially Compressed by A Septal Occluder AV = atrioventricular; LAD = left anterior descending artery; LCx = left circumflex artery; RCA = eight coronary artery.

**Figure 3:**
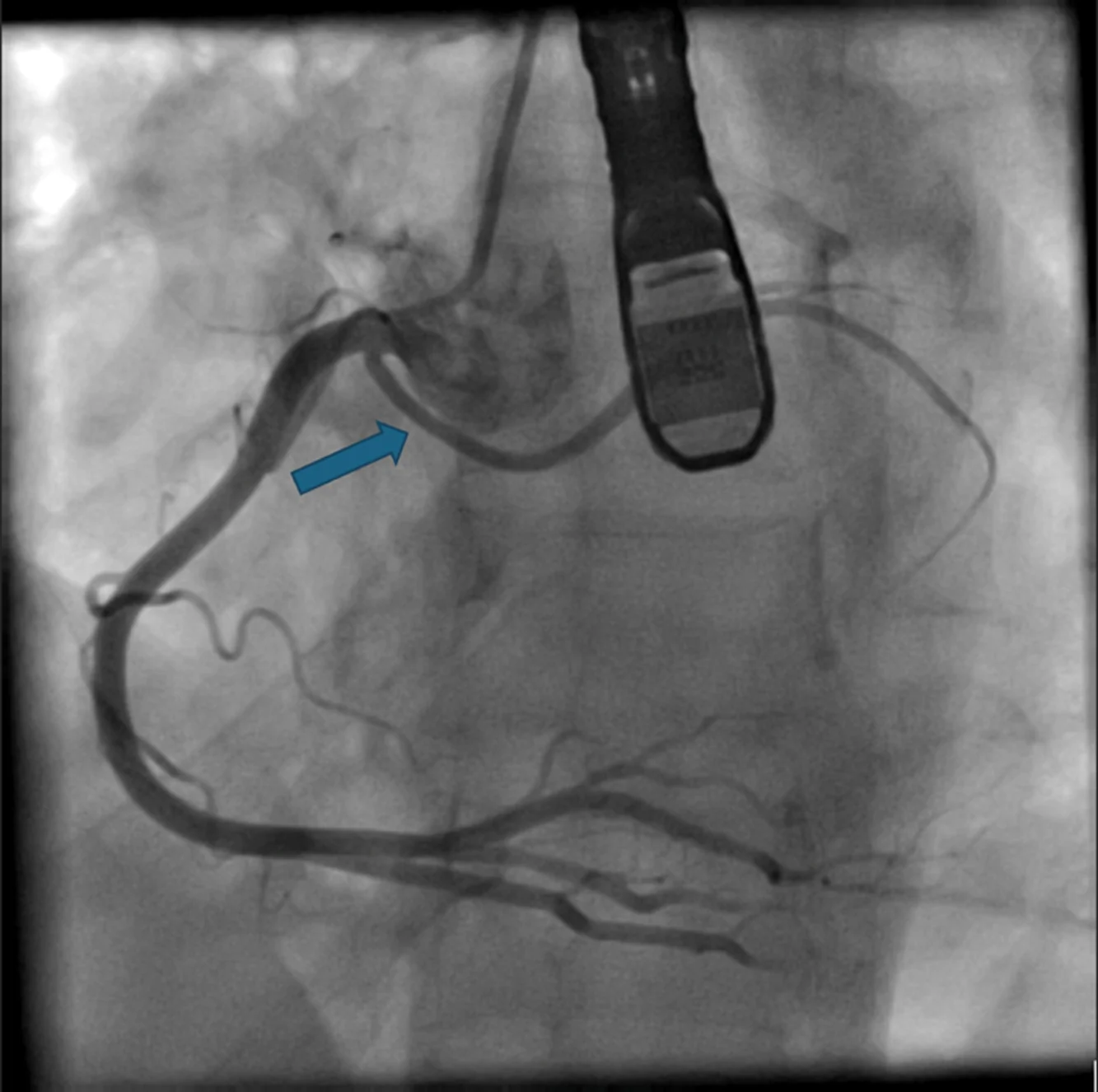
Baseline Coronary Angiography Showing Anomalous Origin of the Left Circumflex Artery From the Right Coronary Artery (Blue Arrow)

The PFO was successfully closed using a 27-mm Occlutech device, with optimal positioning and no residual shunt. Continuous electrocardiographic monitoring showed sinus rhythm without ischemic changes.

Before final device release, repeat RCA angiography confirmed patency of the anomalous LCx with TIMI III flow and no evidence of mechanical compression (Figure 4). The device was then successfully released.

**Figure 4:**
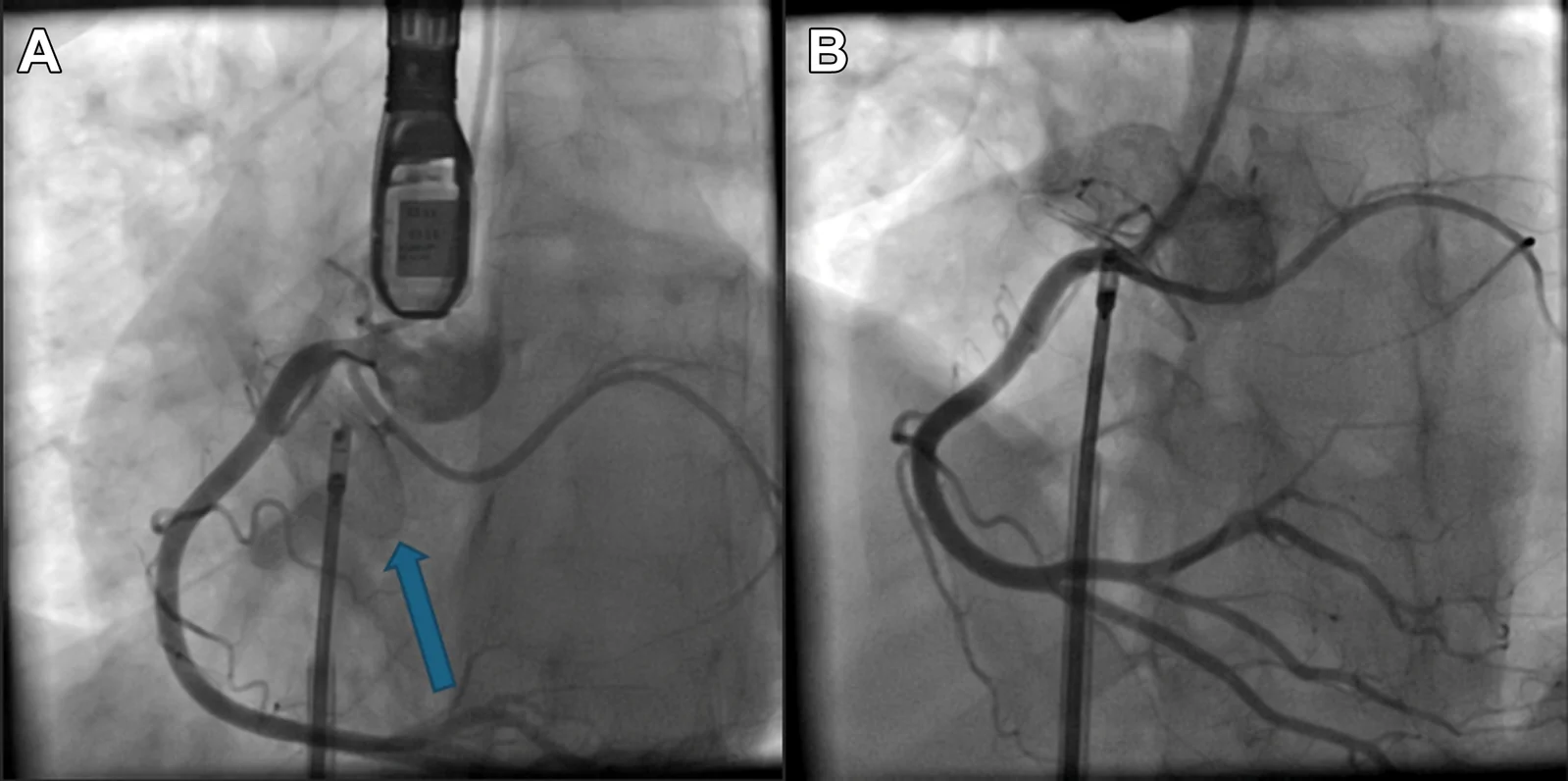
Selective Coronary Angiography at Time of Patent Foramen Ovale Closure (A) Coronary angiography showed patent left circumflex artery with normal flow before the final release of a patent foramen ovale occluder device (blue arrow). (B) Coronary angiography showed stable patent left circumflex artery with normal flow after the final release of the patent foramen ovale occluder device.

The patient remained hemodynamically stable and asymptomatic and was discharged home the following day on a short course of dual antiplatelet therapy.

## Discussion

Transcatheter PFO closure is a safe and widely performed procedure with low complication rates. Acute myocardial ischemia is rare, except in unusual circumstances such as air embolism or mechanical compression of an anomalous coronary artery.^3^

Only 2 case reports have specifically addressed mechanical compression of anomalous coronary arteries during PFO closure.^3^^,^^4^

Casolo et al^3^ reported a unique case of myocardial ischemia due to the compression of an anomalous LCx arising from the right sinus of Valsalva. It was hypothesized that the LCx was compressed between the PFO occluder device and the aortic root.^3^ To mitigate this risk, selection of the smallest closure device capable of effectively sealing the PFO without compromising adjacent structures is recommended.^4^

Miura et al^4^ described a case in which careful preprocedural planning was undertaken to avoid coronary compression. Coronary computed tomography angiography (CCTA) demonstrated an anomalous left coronary artery from the noncoronary cusp, with concern of compression of the single sinus node branch. Risk mitigation strategies included CCTA-based sizing to decide on maximum size allowed, balloon sizing, serial angiography, and intravascular ultrasound of the artery of interest before and after device deployment and before final release.^4^

Although the risk of compression appears low with PFO devices, atrial septal defects (ASD) closure may carry higher risk because of larger defect sizes and device requirement, as well as high-risk anatomical features such as deficient aortic rim. Several case reports have demonstrated safe transcatheter ASD closure in the presence of coronary anomalies.^5^, ^6^, ^7^, ^8^ These included various types of coronary anomalies, most commonly anomalous LCx from RCA or right sinus of Valsalva with retro-aortic course. Other types included single coronary artery arising from the left sinus of Valsalva with the RCA having a retro-aortic course, and a single sinoatrial node artery from the LCx with a retro-aortic course. Device sizes ranged from 18 to 34 mm and preprocedural CCTA planning was crucial in most cases. All cases demonstrated the importance of coronary angiography post device deployment to confirm the absence of compression before final release.

Nevertheless, other case reports have demonstrated safe retrieval of devices before release when anomalous coronary compression was detected angiographically.^9^^,^^10^ The 2 types of anomalies described were a single coronary artery and a left coronary artery both originating from the right sinus of Valsalva. The sizes of the devices used were 12 mm and 24 mm. Patients were then treated surgically with a patch.

In cases in which myocardial ischemia occurs during the procedure, device retrieval should be considered, along with evaluation for alternative surgical options for PFO closure. Surgical approach might include intervention to the anomalous coronary based on the anatomy, origin, and course of the vessel and the clinical context.^1^

In our case, careful intraprocedural assessment confirmed preserved coronary flow and absence of mechanical compression before device release.

Although coronary angiography remains the gold standard for assessment of coronary disease, it is not routinely performed before PFO closure.^2^ There are no consensus recommendations regarding routine coronary assessment before transcatheter PFO closure. In our practice, coronary angiography is performed before PFO closure in patients older than 40 years, those with cardiovascular risk factors, or those presenting with angina. However, anomalous coronary arteries will be missed in some cases. Although rare, these anomalies can potentially result in life-threatening device-related coronary compression leading to myocardial ischemia. One study found that routine coronary angiography performed at time of PFO closure in asymptomatic patients revealed higher than expected prevalence of coronary artery disease (approximately 29%), although severe coronary artery disease was identified in only 9% of patients.^11^ Although the detection of asymptomatic coronary artery disease may not alter the need for coronary revascularization, it could influence primary prevention strategies, including longer duration of antiplatelet and aggressive lipid-lowering therapies. In addition, invasive or noninvasive coronary assessment may identify anomalous coronary arteries with a retro-aortic course, which may be at risk of mechanical compression by the PFO closure device. Noninvasive imaging modalities such as 3-dimensional CCTA and cardiac magnetic resonance imaging can accurately delineate coronary anatomy and may assist in procedural planning, including appropriate device size selection, to minimize the risk of coronary compression.^2^^,^^4^^,^^12^

In summary, the main factors that increase the risk of mechanical compression of an anomalous coronary artery during transcatheter PFO or ASD closure include a retro-aortic anomalous coronary course, a deficient aortic rim in the setting of ASD, and the use of larger closure devices, which are more commonly required for ASD closure. When an anomalous coronary artery is identified, coronary angiography before final device release is essential to confirm coronary patency and exclude device-related compression. If coronary compression is detected, the device should be recaptured and removed, and the patient should be referred for surgical repair. Further studies are needed to determine whether routine coronary assessment before transcatheter PFO closure should be considered in all patients.

## Conclusions

This case highlights the importance of coronary artery assessment before and during transcatheter PFO closure in the presence of coronary anomaly. In patients with retro-aortic coronary variants, reassessment of coronary flow before final device release is essential to exclude mechanical compression and prevent myocardial ischemia.

## Funding Support and Author Disclosures

Dr Al-Bawardy has received consulting/honoraria fees from Amgen, Boston Scientific, Medtronic, Meril, Terumo, and Abbott. All other authors have reported that they have no relationships relevant to the contents of this paper to disclose.

## Appendix

Video 1 A Video Clip of Intraprocedural TEE Including Color Doppler Demonstrates RAC Sign
